# *rac*,*endo*-9-Hy­droxy-9-methyl­tri­cyclo­[5.2.2.0^2,6^]undeca-4,10-dien-8-one

**DOI:** 10.1107/S2414314626008825

**Published:** 2026-08-28

**Authors:** Heiner Detert, Badr El Kadi, Dieter Schollmeyer

**Affiliations:** a University of Mainz, Department of Chemistry, Duesbergweg 10-14, 55099 Mainz, Germany; Goethe-Universität Frankfurt, Germany

**Keywords:** crystal structure, hydrogen bridges, ketol

## Abstract

The tricyclic mol­ecule of the title compound, C_12_H_14_O_2_, is composed of four nearly planar subunits. Hydrogen bonds connect the ketol units to chains along the *a*-axis direction. The structure is further consolidated by two C—H⋯O hydrogen bonds, one to the carbonyl and one to the hy­droxy group.

## Abstract 2

The tricyclic mol­ecule of the title compound is composed of four nearly planar subunits. Hydrogen bonds connect the ketol units to chains along the *a*-axis direction. The structure is further consolidated by two C—H⋯O hydrogen bonds, one to the carbonyl and one to the hy­droxy group.

## Graphical abstract

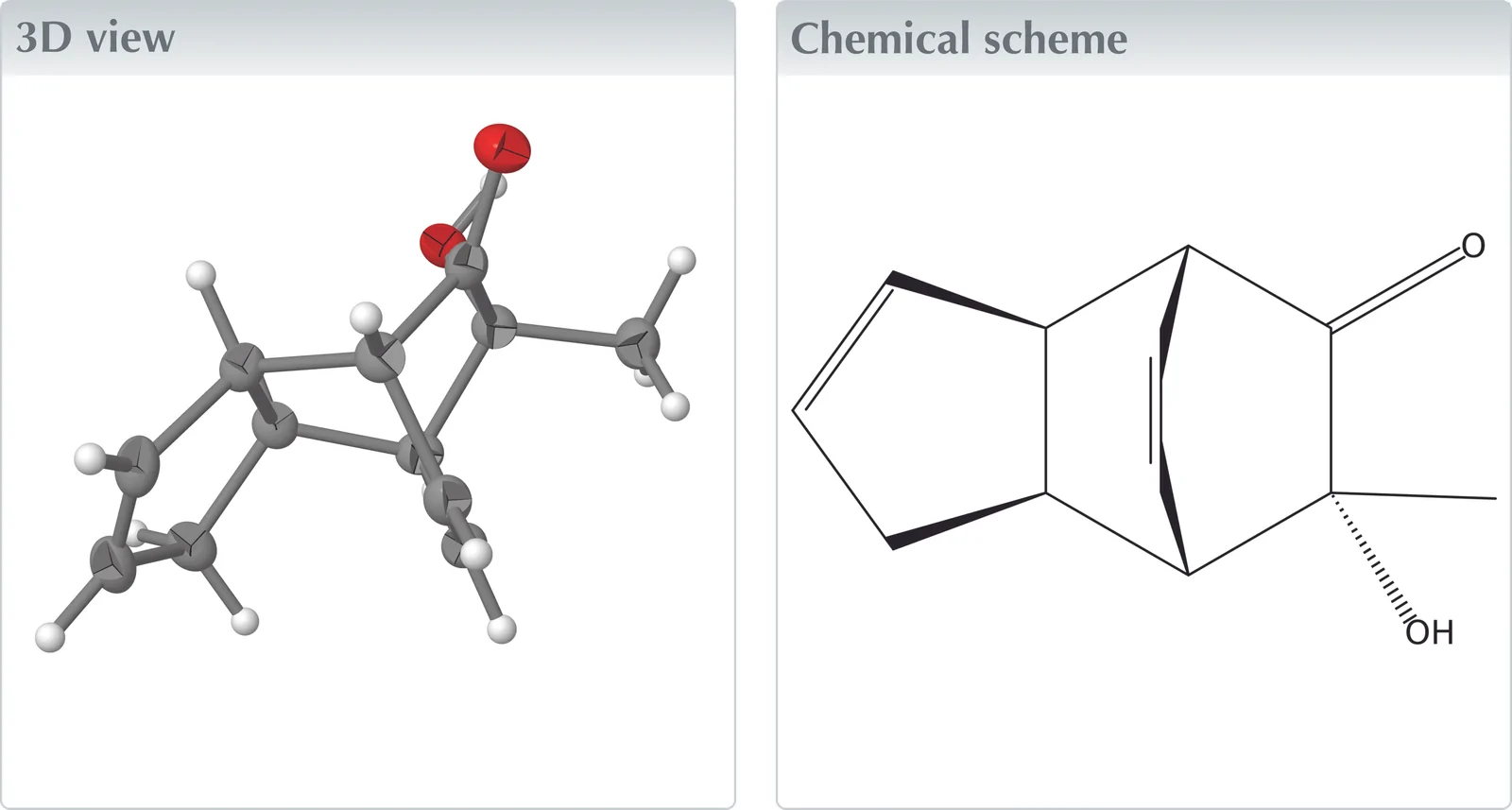

## Structure description

**Figure 1:**
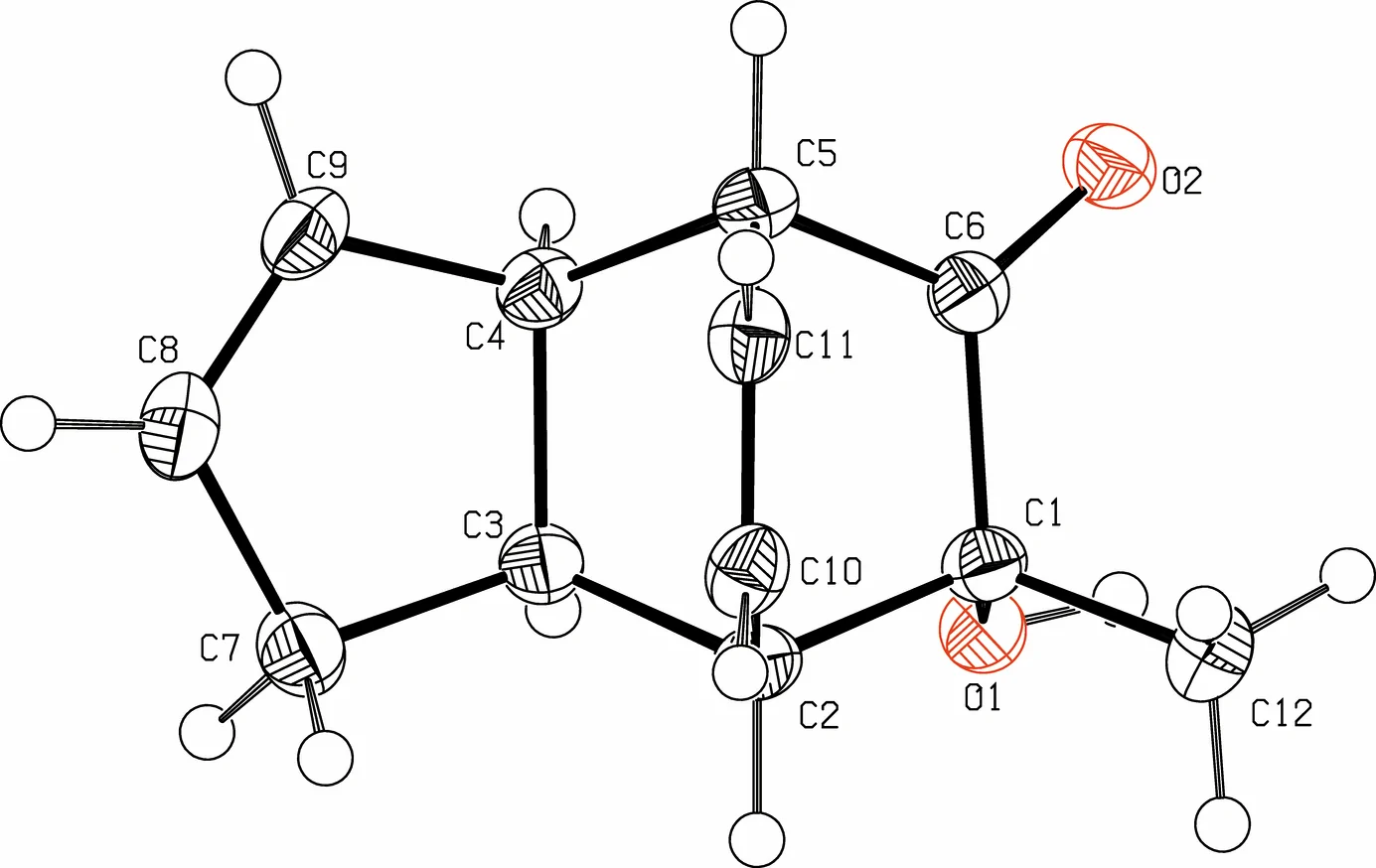
View of the title compound. Displacement ellipsoids are drawn at the 50% probability level.

**Figure 2:**
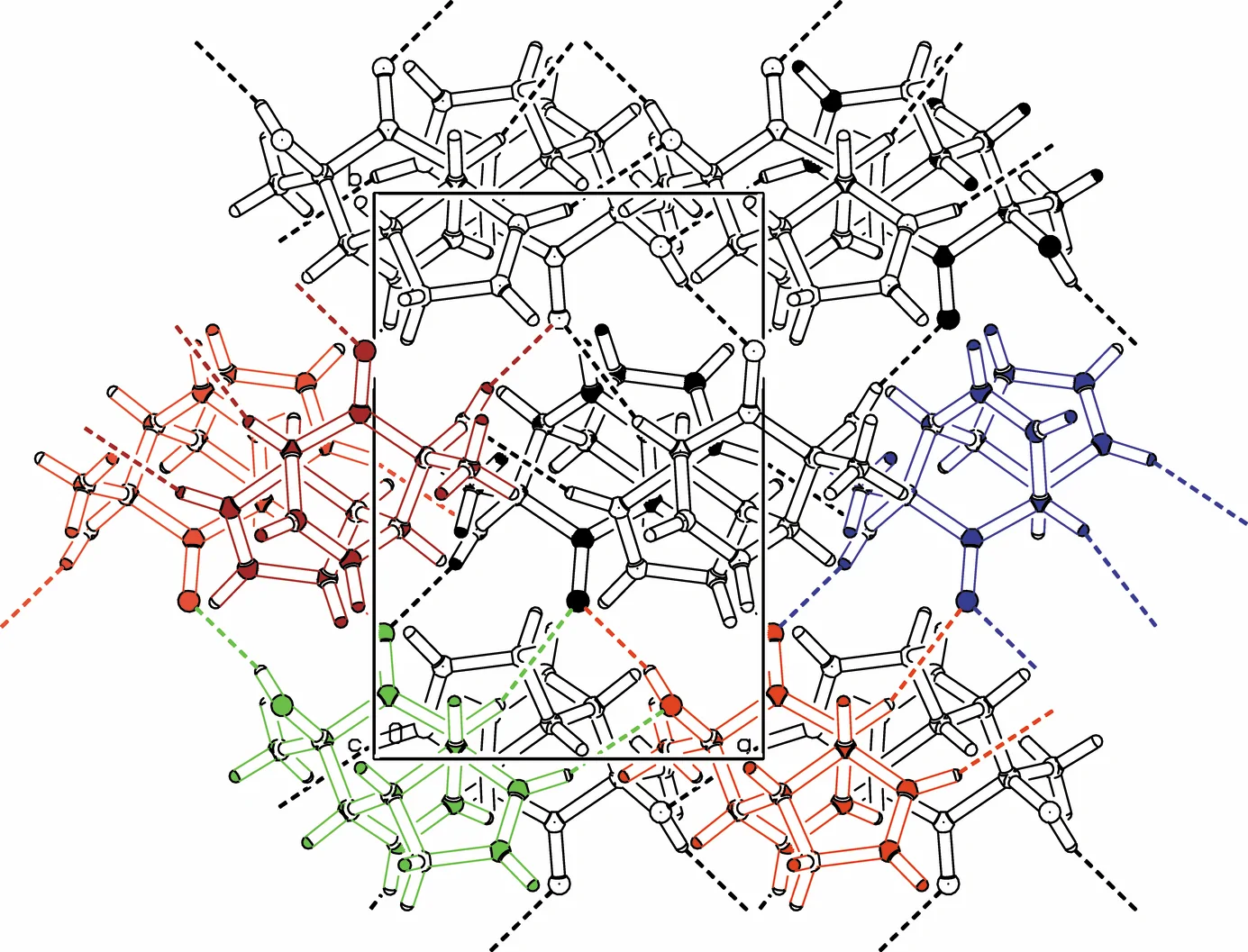
Part of the packing diagram. Hydrogen bonds are shown as dashed lines. View along the *c*-axis direction. Hydrogen atoms not involved in hydrogen bonds are omitted for clarity. Symmetry-related mol­ecules are shown in different colors.

The title compound, C_12_H_14_O_2_ (Fig. 1 ▸), was prepared in a project on tricyclic frameworks (Detert & Schollmeyer, 2020 ▸; Schollmeyer & Detert, 2025 ▸, 2026 ▸). The synthesis was performed in a Diels–Alder cyclo­addition of *o*-quinolacetate and cyclo­penta­diene followed by hydrolysis. The tricyclic mol­ecule is composed of a [2.2.2] propellane with an annulated cyclo­pentene. The nearly *C*_3_-symmetrical carbon framework of the propellane is composed of three C_2_ bridges, these are essentially coplanar with the bridgehead carbons C2, C5. Subunit C2–C1–C6–C5 is planar within 0.0284 (9) Å and subtends a dihedral angle of 58.54 (14)° with the unit C2–C10–C11–C5. The latter is planar within 0.006 (2) Å and the dihedral angle to the planar unit C2–C3–C4–C5 (max. deviation: 0.0032 Å) amounts to 58.09 (15)°. The third angle is slightly larger, 63.38 (14)°. The *endo*-configuration is proven by the dihedral angle between the planar cyclo­pentene [max. deviation 0.010 (2) Å] and the vinyl­ene bridge C2–C10–C11–C5 of 63.14 (14)°. Four mol­ecules fill the unit cell: they are symmetrically connected *via* a twofold screw axis parallel to the *a*-axis and physically *via* hydrogen bonds. The O1—H1*O*⋯O2 hydrogen bonds (Table 1 ▸, Fig. 2 ▸) connect the mol­ecules into chains along the *a*-axis direction, and additional C—H⋯O inter­actions link the chains into ribbons.

## Synthesis and crystallization

The synthesis was performed *via* Diels–Alder reaction of 1.10 g (6.62 mmol) 2-methyl-*o*-quinolacetate (Wessely & Sinwel, 1950 ▸) in 10 ml xylenes by dropwise addition of 0.46 g (1.05 eq) cyclo­penta­diene in 1.5 ml xylenes. The mixture was refluxed for 18 h while the color changed from yellow to orange and crystals separated. The solvent was distilled off, the residue was added to a mixture of 10 ml 1 *M* sodium hydroxide in water and 1 ml of methanol. After 16 h, the mixture was heated to 333 K until a brown compound separated. The mixture was acidulated, the precipitate was again dissolved in sodium hydroxide/methanol and reprecipitated by addition of hydro­chloric acid. Single crystals were grown by slow evaporation of a solution of 60 mg of the precipitate in ether. After filtration through cotton, petroleum ether was added until the solution became turbid and again washed with ether. The now clear solution slowly evaporated and single crystals were collected mechanically from the partially coloured mixture. Yield 22 mg of colourless crystals with m.p. 399-402 K. Literature: 68% yield, m.p. 399-401 K. (Metlesics *et al.*, 1958 ▸). IR (ATR): ñ (cm^−1^): 3403 *s*, 3048*w*, 2983*w*, 2965*w*, 2935*w*, 2918*w*, 2895*w*, 2876*m*, 2845*w*, 1717*vs*, 1619*w*, 1441, 1386*m*, 1364*m*, 1355*m*, 1301*s*, 1264*vw*, 1234*w*, 1200*w*, 1151*s*, 1138*s*, 1112*s*, 1090*m*, 1050, 1014*s*, 975*m*, 948*m*, 925*w*, 892*s*, 853*m*, 790*m*, 778*m*, 750*s*, 731*m*, 708*vs*, 675*s*, 650*w*, 582*vs*, 499*s*; ^1^H-NMR (400 MHz, CDCl_3_): 6.37-6.24 (*m*, 1H, H8), 6.13-6.01 (*m*, 1H, H-11), 5.70 (*dq*, ^3^*J* = 5.7 Hz, ^4^*J* = 2.2 Hz, 1H, H-4), 5.44 (*ddd*, ^3^*J* = 5.8 Hz, ^3^*J* = 3.2 Hz, ^4^*J* = 1.5 Hz, 1H, H-5), 3.30-3.15 (*m*, 3H, H-2,-6,-7,), 3.00 (*dt*, ^3^*J* = 6.7 Hz, ^4^*J* = 2.2 Hz, 1H, H-1), 2.63-2.50 (*m*, 2H), 2.06-1.93 (*m*, 1H), 1.28 (*s*, 3H, CH_3_). Annotation of NMR signals follows IUPAC nomenclature.

## Refinement

Crystal data, data collection and structure refinement details are summarized in Table 2 ▸. The structure was refined as a racemic twin.

## Supplementary Material

Crystal structure: contains datablock(s) I, global. DOI: 10.1107/S2414314626008825/bt4205sup1.cif

Structure factors: contains datablock(s) I. DOI: 10.1107/S2414314626008825/bt4205Isup2.hkl

Supporting information file. DOI: 10.1107/S2414314626008825/bt4205Isup3.cml

CCDC reference: 2583168

Additional supporting information:  crystallographic information; 3D view; checkCIF report

## Figures and Tables

**Table 1:** Hydrogen-bond geometry (Å, °)

*D*—H⋯*A*	*D*—H	H⋯*A*	*D*⋯*A*	*D*—H⋯*A*
O1—H1*O*⋯O2^i^	0.85 (3)	1.99 (3)	2.809 (2)	162 (3)
C5—H5⋯O2^ii^	1.00	2.52	3.407 (3)	148
C9—H9⋯O1^iii^	0.95	2.55	3.450 (3)	159

Symmetry codes: (i) 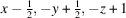; (ii) 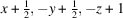; (iii) 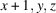.

**Table 2:** Experimental details

Crystal data	
Chemical formula	C_12_H_14_O_2_
*M*_r_	190.23
Crystal system, space group	Orthorhombic, *P*2_1_2_1_2_1_
Temperature (K)	120
*a*, *b*, *c* (Å)	7.3829 (3), 10.7318 (3), 12.2961 (4)
*V* (Å^3^)	974.24 (6)
*Z*	4
Radiation type	Cu *K*α
μ (mm^−1^)	0.70
Crystal size (mm)	0.56 × 0.25 × 0.24

Data collection	
Diffractometer	Stoe Stadivari
No. of measured, independent and observed [*I* > 2σ(*I*)] reflections	5863, 1798, 1754
*R*_int_	0.019
(sin θ/λ)_max_ (Å^−1^)	0.607

Refinement	
*R*[*F*^2^ > 2σ(*F*^2^)], *wR*(*F*^2^), *S*	0.033, 0.091, 1.12
No. of reflections	1798
No. of parameters	134
H-atom treatment	H atoms treated by a mixture of independent and constrained refinement
Δρ_max_, Δρ_min_ (e Å^−3^)	0.30, −0.14
Absolute structure	Refined as an inversion twin
Absolute structure parameter	0.5 (3)

Computer programs: *X-AREA WinXpose*, *X-AREA Recipe* and *X-AREA Integrate* (Stoe & Cie, 2020 ▸), *SHELXT2014* (Sheldrick, 2015*a* ▸), *SHELXL2019/2* (Sheldrick, 2015*b* ▸) and *PLATON* (Spek, 2009 ▸).

## full crystallographic data

### rac,endo-9-Hydroxy-9-methyltricyclo[5.2.2.02,6]undeca-4,10-dien-8-one Crystal data

**Table:**

C_12_H_14_O_2_	*D*_x_ = 1.297 Mg m^−^^3^
*M**_r_* = 190.23	Cu *K*α radiation, λ = 1.54178 Å
Orthorhombic, *P*2_1_2_1_2_1_	Cell parameters from 11043 reflections
*a* = 7.3829 (3) Å	θ = 3.6–69.9°
*b* = 10.7318 (3) Å	µ = 0.70 mm^−^^1^
*c* = 12.2961 (4) Å	*T* = 120 K
*V* = 974.24 (6) Å^3^	Block, colorless
*Z* = 4	0.56 × 0.25 × 0.24 mm
*F*(000) = 408	

### rac,endo-9-Hydroxy-9-methyltricyclo[5.2.2.02,6]undeca-4,10-dien-8-one Data collection

**Table:**

Stoe Stadivari diffractometer	1754 reflections with *I* > 2σ(*I*)
Radiation source: microfocus tube	*R*_int_ = 0.019
Detector resolution: 13.33 pixels mm^-1^	θ_max_ = 69.5°, θ_min_ = 5.5°
rotation method, ω scans	*h* = −8→8
5863 measured reflections	*k* = −13→11
1798 independent reflections	*l* = −14→14

### rac,endo-9-Hydroxy-9-methyltricyclo[5.2.2.02,6]undeca-4,10-dien-8-one Refinement

**Table:**

Refinement on *F*^2^	H atoms treated by a mixture of independent and constrained refinement
Least-squares matrix: full	*w* = 1/[σ^2^(*F*_o_^2^) + (0.0498*P*)^2^ + 0.2628*P*] where *P* = (*F*_o_^2^ + 2*F*_c_^2^)/3
*R*[*F*^2^ > 2σ(*F*^2^)] = 0.033	(Δ/σ)_max_ < 0.001
*wR*(*F*^2^) = 0.091	Δρ_max_ = 0.30 e Å^−^^3^
*S* = 1.12	Δρ_min_ = −0.14 e Å^−^^3^
1798 reflections	Extinction correction: SHELXL2019/2 (Sheldrick, 2015b), Fc^*^=kFc[1+0.001xFc^2^λ^3^/sin(2θ)]^-1/4^
134 parameters	Extinction coefficient: 0.027 (2)
0 restraints	Absolute structure: Refined as an inversion twin
Primary atom site location: dual	Absolute structure parameter: 0.5 (3)
Hydrogen site location: mixed	

### rac,endo-9-Hydroxy-9-methyltricyclo[5.2.2.02,6]undeca-4,10-dien-8-one Special details

**Table:**

Geometry. All esds (except the esd in the dihedral angle between two l.s. planes) are estimated using the full covariance matrix. The cell esds are taken into account individually in the estimation of esds in distances, angles and torsion angles; correlations between esds in cell parameters are only used when they are defined by crystal symmetry. An approximate (isotropic) treatment of cell esds is used for estimating esds involving l.s. planes.
Refinement. Refined as a 2-component inversion twin. The hydroxyl H atom was freely refined. Hydrogen atoms attached to carbons were placed at calculated positions and were refined in the riding-model approximation with C_tertiary_–H = 1.00 Å, C_secondary_–H = 0.99 Å, C_methyl_–H = 0.98 Å, C*_sp_*^2^–H = 0.95 Å, and with *U*_iso_(H) = 1.5 *U*_eq_(C_methyl_) or with *U*_iso_(H) = 1.2 *U*_eq_(C) for the remaining H atoms. The methyl group was allowed to rotate but not to tip.

### rac,endo-9-Hydroxy-9-methyltricyclo[5.2.2.02,6]undeca-4,10-dien-8-one Fractional atomic coordinates and isotropic or equivalent isotropic displacement parameters (Å^2^)

**Table:**

	*x*	*y*	*z*	*U*_iso_*/*U*_eq_	
O1	0.2635 (2)	0.40578 (15)	0.39744 (12)	0.0250 (4)	
H1O	0.210 (4)	0.346 (3)	0.429 (2)	0.044 (9)*	
O2	0.5241 (2)	0.27907 (14)	0.52851 (13)	0.0278 (4)	
C1	0.3673 (3)	0.46715 (19)	0.47879 (17)	0.0224 (5)	
C2	0.4350 (3)	0.5909 (2)	0.43046 (17)	0.0240 (5)	
H2	0.331230	0.647713	0.414050	0.029*	
C3	0.5447 (3)	0.56246 (19)	0.32649 (17)	0.0232 (5)	
H3	0.466378	0.516511	0.273651	0.028*	
C4	0.7112 (3)	0.4804 (2)	0.35644 (16)	0.0230 (5)	
H4	0.706449	0.399843	0.315708	0.028*	
C5	0.7129 (3)	0.45495 (18)	0.48239 (16)	0.0227 (5)	
H5	0.820256	0.404831	0.505697	0.027*	
C6	0.5363 (3)	0.38798 (19)	0.50245 (15)	0.0217 (5)	
C7	0.6217 (3)	0.6810 (2)	0.27121 (19)	0.0291 (5)	
H7A	0.578751	0.687741	0.195158	0.035*	
H7B	0.585296	0.756903	0.311409	0.035*	
C8	0.8233 (3)	0.6633 (2)	0.27516 (18)	0.0294 (5)	
H8	0.907499	0.722808	0.248544	0.035*	
C9	0.8712 (3)	0.5560 (2)	0.31960 (18)	0.0280 (5)	
H9	0.993364	0.529570	0.327363	0.034*	
C10	0.5610 (3)	0.64894 (19)	0.51272 (18)	0.0252 (5)	
H10	0.540961	0.729378	0.542666	0.030*	
C11	0.7019 (3)	0.5787 (2)	0.53996 (17)	0.0248 (5)	
H11	0.789473	0.604630	0.591891	0.030*	
C12	0.2556 (3)	0.4843 (2)	0.58199 (18)	0.0295 (5)	
H12A	0.144834	0.530609	0.564830	0.044*	
H12B	0.326631	0.530835	0.635710	0.044*	
H12C	0.223618	0.402526	0.611783	0.044*	

### rac,endo-9-Hydroxy-9-methyltricyclo[5.2.2.02,6]undeca-4,10-dien-8-one Atomic displacement parameters (Å^2^)

**Table:**

	*U*^11^	*U*^22^	*U*^33^	*U*^12^	*U*^13^	*U*^23^
O1	0.0209 (8)	0.0261 (8)	0.0279 (8)	−0.0035 (6)	−0.0034 (6)	0.0023 (6)
O2	0.0274 (8)	0.0234 (8)	0.0325 (8)	0.0008 (6)	−0.0017 (7)	0.0033 (6)
C1	0.0192 (10)	0.0224 (10)	0.0255 (10)	0.0013 (8)	−0.0007 (8)	0.0000 (8)
C2	0.0202 (10)	0.0216 (10)	0.0302 (11)	0.0021 (8)	−0.0001 (8)	−0.0002 (9)
C3	0.0201 (10)	0.0262 (11)	0.0231 (10)	−0.0008 (8)	−0.0030 (8)	0.0018 (8)
C4	0.0217 (10)	0.0251 (10)	0.0223 (10)	0.0015 (9)	0.0004 (8)	−0.0020 (8)
C5	0.0183 (10)	0.0246 (10)	0.0252 (10)	0.0019 (8)	−0.0016 (8)	0.0013 (8)
C6	0.0224 (10)	0.0252 (11)	0.0174 (9)	0.0005 (9)	−0.0010 (8)	−0.0010 (8)
C7	0.0291 (12)	0.0288 (11)	0.0294 (11)	−0.0026 (9)	−0.0015 (9)	0.0048 (9)
C8	0.0268 (12)	0.0379 (13)	0.0236 (10)	−0.0081 (10)	0.0019 (9)	0.0009 (9)
C9	0.0213 (11)	0.0389 (13)	0.0237 (10)	0.0015 (9)	0.0037 (9)	−0.0026 (9)
C10	0.0250 (10)	0.0242 (10)	0.0265 (11)	−0.0030 (9)	0.0042 (8)	−0.0037 (8)
C11	0.0241 (10)	0.0279 (11)	0.0223 (10)	−0.0054 (8)	0.0000 (8)	−0.0014 (8)
C12	0.0260 (11)	0.0326 (12)	0.0301 (11)	−0.0018 (9)	0.0058 (9)	−0.0019 (9)

### rac,endo-9-Hydroxy-9-methyltricyclo[5.2.2.02,6]undeca-4,10-dien-8-one Geometric parameters (Å, º)

**Table:**

O1—C1	1.422 (3)	C5—C11	1.508 (3)
O1—H1O	0.85 (3)	C5—C6	1.509 (3)
O2—C6	1.215 (3)	C5—H5	1.0000
C1—C12	1.524 (3)	C7—C8	1.501 (3)
C1—C6	1.538 (3)	C7—H7A	0.9900
C1—C2	1.538 (3)	C7—H7B	0.9900
C2—C10	1.509 (3)	C8—C9	1.323 (3)
C2—C3	1.544 (3)	C8—H8	0.9500
C2—H2	1.0000	C9—H9	0.9500
C3—C7	1.550 (3)	C10—C11	1.328 (3)
C3—C4	1.556 (3)	C10—H10	0.9500
C3—H3	1.0000	C11—H11	0.9500
C4—C9	1.503 (3)	C12—H12A	0.9800
C4—C5	1.573 (3)	C12—H12B	0.9800
C4—H4	1.0000	C12—H12C	0.9800
			
C1—O1—H1O	106 (2)	C6—C5—H5	112.5
O1—C1—C12	110.50 (17)	C4—C5—H5	112.5
O1—C1—C6	108.33 (16)	O2—C6—C5	124.45 (19)
C12—C1—C6	110.38 (17)	O2—C6—C1	121.39 (19)
O1—C1—C2	107.65 (16)	C5—C6—C1	114.02 (16)
C12—C1—C2	113.15 (17)	C8—C7—C3	104.18 (18)
C6—C1—C2	106.63 (16)	C8—C7—H7A	110.9
C10—C2—C1	107.33 (17)	C3—C7—H7A	110.9
C10—C2—C3	108.26 (18)	C8—C7—H7B	110.9
C1—C2—C3	108.67 (17)	C3—C7—H7B	110.9
C10—C2—H2	110.8	H7A—C7—H7B	108.9
C1—C2—H2	110.8	C9—C8—C7	112.9 (2)
C3—C2—H2	110.8	C9—C8—H8	123.5
C2—C3—C7	113.16 (18)	C7—C8—H8	123.5
C2—C3—C4	109.28 (17)	C8—C9—C4	112.6 (2)
C7—C3—C4	106.19 (18)	C8—C9—H9	123.7
C2—C3—H3	109.4	C4—C9—H9	123.7
C7—C3—H3	109.4	C11—C10—C2	114.70 (19)
C4—C3—H3	109.4	C11—C10—H10	122.7
C9—C4—C3	104.15 (17)	C2—C10—H10	122.7
C9—C4—C5	112.61 (18)	C10—C11—C5	115.05 (19)
C3—C4—C5	109.73 (17)	C10—C11—H11	122.5
C9—C4—H4	110.1	C5—C11—H11	122.5
C3—C4—H4	110.1	C1—C12—H12A	109.5
C5—C4—H4	110.1	C1—C12—H12B	109.5
C11—C5—C6	107.27 (16)	H12A—C12—H12B	109.5
C11—C5—C4	107.98 (16)	C1—C12—H12C	109.5
C6—C5—C4	103.71 (16)	H12A—C12—H12C	109.5
C11—C5—H5	112.5	H12B—C12—H12C	109.5
			
O1—C1—C2—C10	−174.12 (16)	C11—C5—C6—C1	49.8 (2)
C12—C1—C2—C10	63.5 (2)	C4—C5—C6—C1	−64.3 (2)
C6—C1—C2—C10	−58.0 (2)	O1—C1—C6—O2	−55.1 (2)
O1—C1—C2—C3	−57.2 (2)	C12—C1—C6—O2	66.0 (2)
C12—C1—C2—C3	−179.65 (18)	C2—C1—C6—O2	−170.69 (18)
C6—C1—C2—C3	58.8 (2)	O1—C1—C6—C5	120.74 (18)
C10—C2—C3—C7	−63.2 (2)	C12—C1—C6—C5	−118.15 (19)
C1—C2—C3—C7	−179.44 (17)	C2—C1—C6—C5	5.1 (2)
C10—C2—C3—C4	54.9 (2)	C2—C3—C7—C8	118.3 (2)
C1—C2—C3—C4	−61.4 (2)	C4—C3—C7—C8	−1.6 (2)
C2—C3—C4—C9	−121.26 (19)	C3—C7—C8—C9	1.6 (3)
C7—C3—C4—C9	1.1 (2)	C7—C8—C9—C4	−1.0 (3)
C2—C3—C4—C5	−0.5 (2)	C3—C4—C9—C8	−0.1 (2)
C7—C3—C4—C5	121.89 (18)	C5—C4—C9—C8	−119.0 (2)
C9—C4—C5—C11	62.1 (2)	C1—C2—C10—C11	58.2 (2)
C3—C4—C5—C11	−53.4 (2)	C3—C2—C10—C11	−58.9 (2)
C9—C4—C5—C6	175.74 (17)	C2—C10—C11—C5	1.1 (3)
C3—C4—C5—C6	60.2 (2)	C6—C5—C11—C10	−55.4 (2)
C11—C5—C6—O2	−134.5 (2)	C4—C5—C11—C10	55.8 (2)
C4—C5—C6—O2	111.4 (2)		

### rac,endo-9-Hydroxy-9-methyltricyclo[5.2.2.02,6]undeca-4,10-dien-8-one Hydrogen-bond geometry (Å, º)

**Table:**

*D*—H···*A*	*D*—H	H···*A*	*D*···*A*	*D*—H···*A*
O1—H1*O*···O2^i^	0.85 (3)	1.99 (3)	2.809 (2)	162 (3)
C5—H5···O2^ii^	1.00	2.52	3.407 (3)	148
C9—H9···O1^iii^	0.95	2.55	3.450 (3)	159

Symmetry codes: (i) *x*−1/2, −*y*+1/2, −*z*+1; (ii) *x*+1/2, −*y*+1/2, −*z*+1; (iii) *x*+1, *y*, *z*.
